# Features of architectural landscape fragmentation in traditional villages in Western Hunan, China

**DOI:** 10.1038/s41598-023-45099-y

**Published:** 2023-10-30

**Authors:** Can Zeng, Peilin Liu, Liuqian Huang, Shun Feng, Yu Li

**Affiliations:** 1 College of Geographical Sciences, Hunan Normal University, Changsha, 410081 Hunan China; 2 UNESCO International Centre for HIST Changsha Workstation, Changsha, 410022 Hunan China; 3 College of Architecture and Urban Planning, Guangdong University of Technology, Guangzhou, 510090 Guangdong China; 4 Hunan Vocational College of Technology, Changsha, 410001 Hunan China; 5 State Environmental Protection Key Laboratory of Urban Ecological Simulation and Protection, South China Institute of Environmental Sciences, Ministry of Ecology and Environment, Guangzhou, 510655 China

**Keywords:** Ecology, Environmental social sciences

## Abstract

With rapid industrialization and urbanization in China, inadequate preservation of traditional architecture coupled with natural deterioration have led to the fragmentation of architectural landscapes. Drawing from ecological fragmentation research in landscape ecology, we consider the cultural landscape as our research object, viewing buildings as landscape patches, and determine a system for measuring architectural landscape fragmentation in traditional villages. The study shows the degree of landscape fragmentation can reveal the characteristics of traditional villages and the process of regional modernization. The results are as follows: (1) From the perspective of landscape diversity, the study area was rich in landscape types in all dimensions, and the relative evenness index was high, signifying evident or severe fragmentation. (2) The index of landscape heterogeneity in the dimensions of building quality, height, and landscape appearance is low in the study area, with mild levels of landscape fragmentation caused by heterogeneity in the aforementioned dimensions. (3) Mild fragmentation suggests the integrity and homogeneity of architectural landscape types, reflecting a lagging level of economic development, whereas high fragmentation signifies rapid economic development, leading to a substantial deterioration in the integrity and homogeneity of architectural landscape types. Therefore, efforts to preserve and develop traditional villages should not solely aim for low fragmentation as it could potentially constrain sustainable development.

## Introduction

Traditional Chinese villages exemplify the successful active adaptation to, and effective use of, the environment, including environmental protection^1^. As the cradle of Chinese farming civilization, traditional villages hold immense historical, cultural, aesthetic, and economic values enjoying high esteem and protection from the government^2–4^. The traditional village landscape is a unique symbol reflecting regional and cultural landscape differences. Traditional Chinese architectural landscapes, shaped by inhabitants with unique geographic and environmental mindsets, incorporate factors such as the natural environment, economic conditions, customs, and ethical values of their long-standing lifestyles. They bear distinct regional identities and offer an unparalleled sense of place. They are considered ideal and natural heritage transmitters and express China’s national characteristics and rich history of traditional culture, seated at the heart of a village’s cultural landscape. Generally, traditional Chinese architecture refers to architecture from the pre-Qin dynasty to the mid-nineteenth century and represents an independently formed architectural system.

However, rapid industrialization and urbanization have undermined the preservation and development of traditional villages. Environmental impacts, the trend towards replacing older buildings, informal construction, infrastructure-related construction, and commercial expansion have all threatened traditional villages, resulting in the fracturing and fragmentation of traditional village architectural landscapes. This scenario poses a significant threat to the protection, transmission, and effective utilization of unique Chinese, vernacular, and cultural genes^5^. Therefore, it is crucial to address the fragmentation of traditional village architectural landscapes into ‘patches’ or ‘islands’^6,7^. Ensuring the organic transmission and integration of landscape genes is essential for the ongoing renewal of architectural landscapes.

Landscape fragmentation refers to the gradual changes wherein previously coherent landscape elements become discontinuous or transform into patchy mosaics due to disturbances caused by anthropogenic effects^8^. Landscape indices are typically used in quantitative studies to describe landscape patterns and their changes. Recent research has mostly analyzed landscape fragmentation within the scope of landscape ecology, focusing on topics such as land use analysis and construction, vegetation cover changes, landscape patterns^9–12^, ecosystem sensitivity evaluations, ecosystem service value analysis, and ecological security network pattern construction^13–16^. The studies also include the impact of climate change on landscape fragmentation, species evolution^17–19^, regional human landscape characteristics, and their conservation^20–25^. An observable trend has emerged where the analysis has shifted from natural to human landscape ecosystems^26^.

A cultural landscape is a complex blend of natural and human factors that embodies the characteristics of a certain region, continually evolving due to human activities^27,28^. Traditional architecture, such as that found in typical rural villages, represents a cultural landscape under threat of fragmentation, a situation exacerbated by urbanization and the rapid development of rural tourism.

This study aimed to clarify the implications of architectural landscape fragmentation and measure its degree. It questions whether fragmentation was caused by heterogeneous landscapes and if the fragmented architectural landscape is entirely detrimental to the protection, development, and historical value of villages.

Most studies initiate their research by classifying the fragmentation of architectural landscapes based on land type and then analysing their land fragmentation characteristics and spatial heterogeneity features in terms of their morphology. Traditional villages often have rigorous protection mechanisms, and the architecture of their land typically does not undergo significant morphological changes during development. This tendency often leads to overlooking vital aspects. Considering a single building as a landscape patch and studying its fragmentation characteristics in terms of architectural quality, style, age, height, and other intrinsic functions have not been thoroughly covered in the literature^6,7,26^.

Given this background, and borrowing from the theory of landscape ecology, this study constructed an index system and evaluation criteria for architectural landscape fragmentation from the perspectives of landscape diversity and heterogeneity. Traditional villages in Western Hunan were taken as examples, and the fragmentation characteristics of their architectural landscapes were analyzed to provide a reference for traditional villages to define continuous, homogeneous, diversified, and moderately fragmented architectural landscapes.

## Theories and concepts

Landscape ecology views fragmentation as a transformation process wherein landscapes, owing to disturbances from natural or human factors, transition from simple, homogeneous, and coherent wholes to complex, heterogeneous, and discontinuous patchworks or mosaics^29,30^. This transition can be classified into four spatial processes, namely perforation, fragmentation, shrinkage, and disappearance. The analysis of natural landscape fragmentation typically involves three aspects: the division of landscape elements into patches, the heterogeneity of landscape elements, and the spatial interrelationships among landscape elements^31^.

Architecture is the space created for human habitation and use that, composed of building materials such as clay, brick, tile, stone, and wood. Vitruvius, the ancient Roman architect, proposed three criteria for architecture in his classic work, ‘The Ten Books of Architecture’: firmitas (sturdiness), utilitas (utility), and venustas (aesthetics). Traditional Chinese architecture predominately consists of wood-frame buildings, while traditional Western and modern architecture are largely characterized by masonry and reinforced concrete, respectively.

In traditional Chinese villages, architectural landscapes are primarily composed of buildings. However, due to rapid socio-economic development and urbanization, some traditional buildings have been demolished or disappeared, leading to the emergence of heterogeneous architectural landscapes with varied structures and styles, which increases the fragmentation of architectural landscapes.

Three types of landscape fragmentation can be observed in traditional village architecture: (1) self-loss fragmentation, which arises from natural deterioration, such as the collapse of old buildings, resulting in the fragmentation of the overall landscape; (2) foreign-loss fragmentation, which occurs through the replacement of old structures with new ones. When the new style is clashes with the original aesthetic, it leads to fragmentation of the local landscape; and (3) external-loss fragmentation, which emerges from the visual and stylistic incongruity between traditional village buildings and surrounding modern structures, leading to the fragmentation of the peripheral landscape.

If we consider each building as a separate patch and disregard the space between them, the properties of the individual building patches (maintaining their structural integrity to ensure functionality) determine the fragmentation of the building landscape. Single buildings do not result in shape fragmentation, which only occurs in relation to the building landscape types and patterns. Therefore, the fragmentation of architectural landscapes can be studied without analyzing the fragmentation of patches and patch shapes, focusing on two aspects: landscape type fragmentation and landscape space interrelationship fragmentation.

From the perspective of landscape diversity and heterogeneity, landscape fragmentation manifests either through the impact of climatic conditions or human activities, such as urbanization, leading to fragmentation characterized by a decrease in the quality of certain buildings. This fragmentation also appears through challenges faced by traditional buildings attempting to accommodate modern living requirements, for example, in terms of function, including aspects such as functionality, comfort, safety, lighting, and ventilation. This results in alterations to building styles, number of stories, and structures. The fragmentation of building functions often sees residential purposes replaced and commercial buildings added to cater to the evolving needs of rural tourism. Typically, the more varied the landscape type, the higher the degree of fragmentation. For instance, if all buildings are one-story tall, the landscape is homogenous and coherent, with no fragmentation. In contrast, if buildings display a range of landscape types, such as one-, two-, and three-story buildings, the emerging heterogeneity amplifies the complexity and unpredictability of the architectural landscape, thus inducing fragmentation.

**Figure 1:**
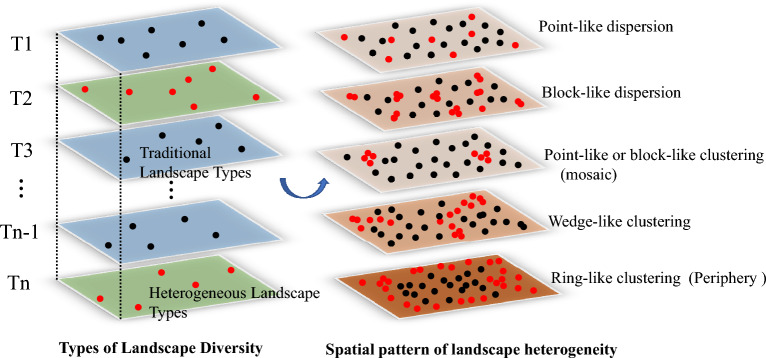
Spatial pattern of diversity and heterogeneity of architectural landscape types.

**Figure 2:**
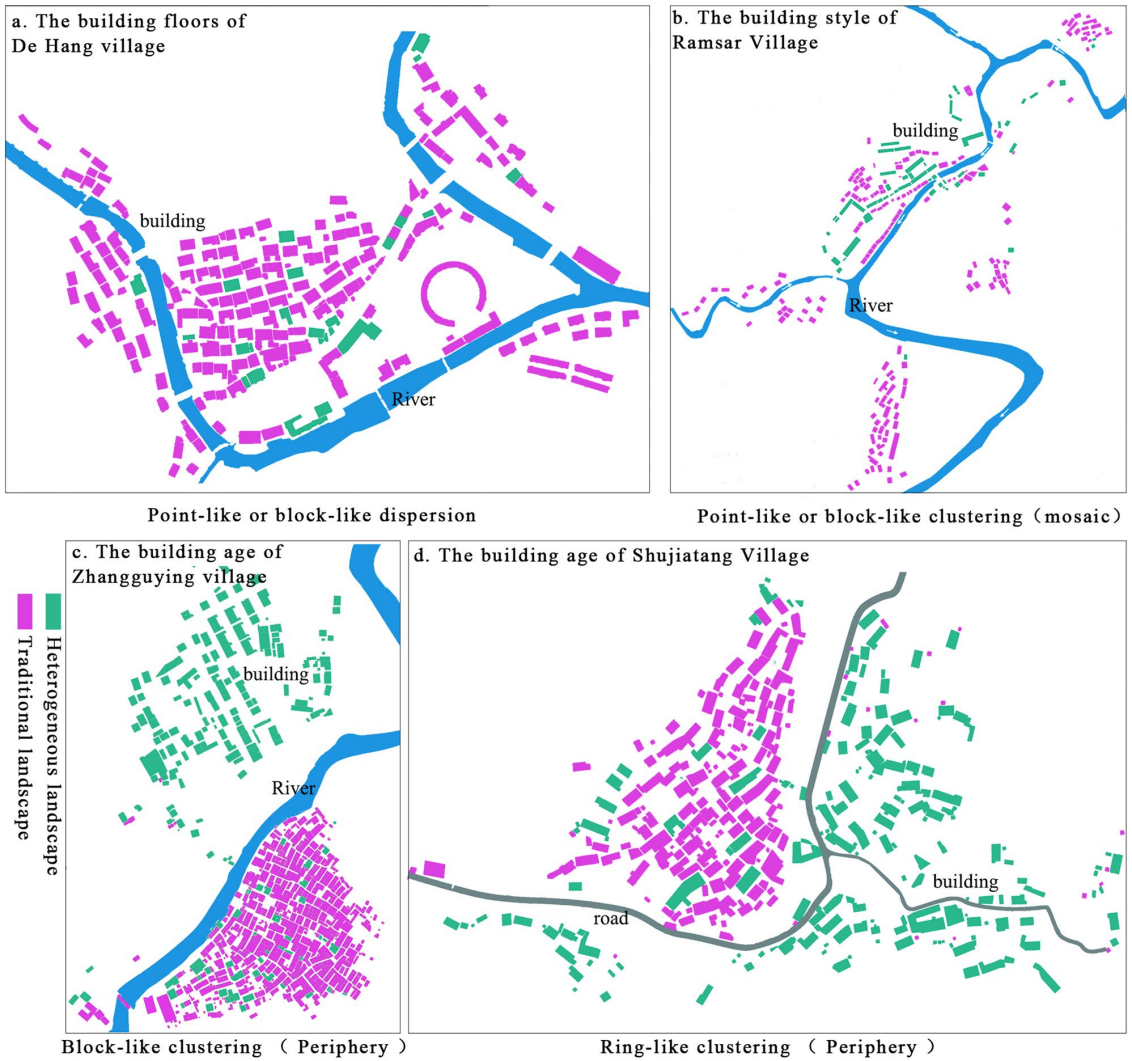
Spatial pattern type of traditional village architectural landscape fragmentation schematic. The author used Adobe Photoshop CS6 software (13.0)^33^ to color-code traditional architectural landscapes and heterogeneous architectural landscapes based on traditional village mapping maps.

Moreover, spatial pattern fragmentation of architectural landscapes refer to the strength of the spatial mosaic created by differing types of architectural landscapes. In traditional landscapes, the extent of heterogeneous agglomeration and dispersion can reveal the type of architectural landscape fragmentation (Fig. 1). When heterogeneous landscapes are clustered, the degree of fragmentation is low when there are fewer landscape types (point-like or block-like clustering). However, if numerous landscape types exist, an ‘islanding’ phenomenon may quickly appear (like wedge-shaped, ring-shaped, or network-like distribution)^32^. External loss fragmentation represents the wider pattern of foreign loss fragmentation. In essence, the more noticeable the external loss fragmentation, the stronger the islanding features of the traditional landscape. When heterogeneous landscapes are scattered, the degree of fragmentation is low if landscape types are few (as in point-like scattered distribution) and high if many landscape types exist (as in block-like scattered distribution) (Fig. 2).

Given its scope, this study emphasizes the fragmentation characteristics of various landscape types within traditional village architecture. Figure 1 shows the different spatial patterns formed as the types and numbers of heterogeneous landscapes increase. Figure 2 presents the spatial patterns formed by the various types of heterogeneous architectural landscapes in typical villages, and it was drawn by Adobe Photoshop CS6 software (13.0)^33^.

## Research methods

### Methodology

Methods for analysing landscape diversity and heterogeneity have been used to study the fragmentation characteristics of various traditional village architectural landscapes. Generally, the landscape diversity index is used to pinpoint the fragmentation of architectural landscape types, while the landscape heterogeneity index is utilized to determine if this fragmentation arises from heterogeneous landscapes^34,35^. Landscape indices discern landscape types and pattern information, thereby mirroring spatial structures and characteristics^36^. The choice of indices should depend on their relevance, calculability, interpretability, and non-redundancy^8^.

Among the numerous indices reflecting the study of landscape diversity and heterogeneity, three-patch multiplicity, diversity, and heterogeneity was selected as the basis for fragmentation analysis. Landscape patch multiplicity serves an auxiliary index, while the landscape diversity index encompasses homogeneity (Simpson’s diversity index) and abundance (Shannon–Wiener diversity index)^37,38^. For analytical convenience, relative homogeneity and abundance indices were employed.

(1) Plaque multiplicity (P_i_)

1 $${\text{P}}_{{\text{i}}} = {\text{n}}_{{\text{i}}} /\Sigma {\text{n}}_{{\text{i}}}$$

where n_i_ is the number of landscape types and Σn_i_ signifies the sum of all landscape types^39^.

(2) Relative homogeneity index (E)

2 $${\text{E}} = {\text{D/D}}_{{{\text{max}}}}$$

where ‘D’ is Simpson’s diversity index (SIDI) and D_max_ symbolizes the maximum possible uniformity under the given landscape type. ‘D’ is more sensitive to the homogeneity of landscape types^40^.

3 $$D = 1 - \sum\limits_{i = 1}^{S} {P_{i}^{2} }$$

where ‘S’ represents the total number of landscape types. The lowest Simpson’s diversity index value was zero, denoting only one unfragmented landscape. When two or more landscape types existed in equal numbers, the ‘D’ value reached its maximum. For instance, with two landscape types, the ‘D’ value was 0.5; with three types, ‘D’ was 0.667; and with more uniform landscape types, ‘D’ was 1, indicating a completely fragmented landscape. When the landscape type was certain, a smaller ‘D’ value indicated less uniformity for each landscape type and a low degree of fragmentation.

(3) Relative abundance index (R)

4 $${\text{R}} = {\text{H/H}}_{{{\text{max}}}}$$

where ‘H’ is the Shannon–Wiener diversity index (SHDI) and H_max_ is the maximum possible richness. An increase in the type of architectural landscape signifies a rise in the complexity of the architectural community; that is, the larger the ‘H’ value, the greater the information content of the architectural landscape and the greater the fragmentation^41^. When only one landscape type was present, the ‘H’ value was zero, indicating no fragmentation. When two or more landscape types were present in equal numbers, the ‘H’ value achieved its maximum.

5 $${\text{H}} = - \sum\limits_{{{\text{i}} = 1}}^{{\text{m}}} {({\text{p}}_{{\text{i}}} )} \log_{2} ({\text{p}}_{{\text{i}}} )$$

In Eq. (5), ‘m’ is the total number of landscape types and P_i_ is the probability of occurrence of a particular landscape type ‘I’.

(4) Landscape heterogeneity index (F)

The heterogeneity index for a traditional village architectural landscape type (F) encapsulates the ratio of the heterogeneous landscape index to the traditional landscape index within an area. When ‘F’ is 0, no heterogeneous landscape or heterogeneous landscape fragmentation is present; when ‘F’ is 1, the quantity of heterogeneous and traditional landscape patches are equal, indicating conspicuous landscape fragmentation. A higher heterogeneity index for traditional village architectural landscape types signifies a stronger impact of modernization on the traditional architectural landscape, leading a higher degree of fragmentation^42^. The calculation is shown below.

6 $${\text{F}} = \Sigma {\text{S}}_{{\text{y}}} /\Sigma {\text{S}}_{{\text{z}}}$$

where ΣS_y_ denotes the sum of the number of patches of heterogeneous landscape types, and ΣS_z_ symbolizes the sum of the number of patches of traditional landscape types^43^.

### Evaluation criteria for traditional village architectural landscape fragmentation

The fragmentation of traditional village architectural landscapes was assessed based on landscape diversity and heterogeneity. Landscape diversity was measured using the relative evenness (E) and relative richness (R) indices. The value ranges of both ‘E’ and ‘R’ were [0, 1], corresponding to the degree of landscape fragmentation. The value ranges were classified into four levels: 0 = no fragmentation; (0, 0.35] = mild fragmentation; (0.35, 0.7] = evident fragmentation; and (0.7, 1] = severe fragmentation. A value of zero signifies the presence of only one landscape type and hence, no fragmentation. A larger ‘E’ value indicates an even distribution of patches across each type of landscape, high landscape complexity, and a high degree of fragmentation. Similarly, a larger ‘R’ value indicates that when the landscape type is fixed, the number of patches across each type of landscape tends to be equal, translating into high landscape complexity, and the high fragmentation. Landscape heterogeneity was primarily evaluated using the heterogeneity index for the traditional village architectural landscape type (F). The value range of ‘F’ is [0, + ∞) corresponding to the degree of landscape fragmentation, and is split into four levels: 0 = no fragmentation; (0, 0.5] = mild fragmentation; (0.5, 1] = evident fragmentation; and (1, + ∞) = severe fragmentation (Table 1).

**Table 1:** Evaluation criteria for the fragmentation of architectural landscape types in traditional villages.

	No fragmentation	Mild fragmentation	Obvious fragmentation	Serious fragmentation
Landscape diversity perspective	0	(0, 0.35]	(0.35, 0.7]	(0.7, 1]
Landscape heterogeneity perspective	0	(0, 0.5]	(0.5, 1]	(1, + ∞)

### Indicators of architecture landscape fragmentation

According to the ‘Basic Requirements for the Preparation of Traditional Village Protection Development Planning’ issued by the Ministry of Housing and Urban–Rural Development (MOHURD) of the People’s Republic of China (PRC) in 2013, the architectural value and characteristics of traditional villages are typically analysed in terms of architectural landscape, architectural age, structure, quality, number of stories, roof style, and architectural functions. Architectural quality can signal the level of ‘self-damaging fragmentation’ of the architectural landscape; architectural style (roof) and structure can denote the level of ‘heterogeneous fragmentation,’ in both external and internal styles; architectural age can reveal the level of ‘heterogeneous fragmentation’ over time; and the number of stories can suggest the level of ‘heterogeneous fragmentation’. The fragmentation of the architectural landscape is characterized by the degree of variation and deterioration of architectural landscape types, combinations, and attributes over space or time. To quantify the degree of fragmentation of architectural landscape types, the existence of traditional and heterogeneous landscapes must be discerned and analysed. For instance, in architectural landscapes, cultural heritage protection units (FM1), historical architecture (FM2), and traditional landscape architecture (FM3) constitute types of traditional village architectural landscapes, while new architecture with incongruent landscapes (FM4) type is considered heterogeneous as it contrasts with the aforementioned traditional landscape types. Based on this, the traditional village architectural landscape was classified into seven major categories and 30 subcategories (Table 2), with each major category further divided into two medium categories: traditional and heterogeneous landscapes.

**Table 2:** Indicators system for measuring the fragmentation of the architectural landscape in traditional villages.

Serial no	Primary indicators	Secondary indicators	Tertiary indicators
1	Architectural style (FM)	Traditional landscape	Cultural Heritage Protection Unit FM1
			Historical architectural FM2
			Traditional style buildings (including new style coordinated buildings) FM3
		Heterogeneous landscape	New architectural with incongruent landscape FM4
2	Architectural age (ND)	Traditional landscape	Ming Dynasty and earlier buildings ND1
			Qing Dynasty Architectural ND2
			Architecture of the Civil War ND3
		Heterogeneous landscape	1950s–1970s architecture ND4
			1980s and later architecture ND5
3	Architectural structure (JG)	Traditional landscape	Rammed earth construction JG1
			Timber frame construction JG2
			Brick and timber construction JG3
		Heterogeneous landscape	Brick and mortar construction JG4
			Other construction JG5
4	Architectural quality (ZL)	Traditional landscape	Good construction quality ZL1
			Average construction quality ZL2
		Heterogeneous landscape	Poor construction quality ZL3
5	Number of floors (GD)	Traditional landscape	One-story building GD1
			Two-story building (including 1.5-story) GD2
		Heterogeneous landscape	Buildings of three-story and above GD3
6	Building roof (WD)	Traditional landscape	Sloped roof buildings (including traditional roof types such as single and double pitched roofs) WD1
		Heterogeneous landscape	Flat roof architecture WD2
			Other buildings (e.g. non-Chinese style) WD3
7	Architecture function (GN)	Traditional landscape	Residential buildings (housing and its ancillary buildings) GN1
			Public-type buildings (such as education, administration, medical, etc.) GN2
			Ritual-type buildings (ancestral shrines, temples, theatres, etc.) GN3
			Defensive buildings (gatehouses, night watch houses, etc.) GN4
		Heterogeneous landscape	Commercial buildings (includes buildings for catering, accommodation, entertainment, management, etc.) GN5
			Commercial and residential buildings (mixed commercial and residential) GN6
			Other functional buildings GN7

## Materials and data

### Study area

Located in the northwestern region of Hunan, Xiangxi Tujia and Miao Autonomous Prefecture are the only autonomous prefectures of ethnic minorities in the province, encompassing one county-level city and seven counties^44^. This study examined Phoenix County, one of the first batch of national historical and cultural cities within this region. Renowned for its abundance natural and cultural heritage sites, this area retains well-preserved villages of the Tujia and Miao ethnic groups, making it a cradle of Wuling culture. Our analysis incorporated 172 villages in Western Hunan from the list of traditional Chinese villages (17, 8, 4, 53, and 90 from the first, second, third, fourth, and fifth batches in 2012, 2013, 2014, 2016, and 2019, respectively), ranking first in Hunan Province and fourth nationally, forming a ‘colony’ of traditional villages. Given the mountainous terrain and abundant timber, wooden houses dominate the residential architecture.

**Figure 3:**
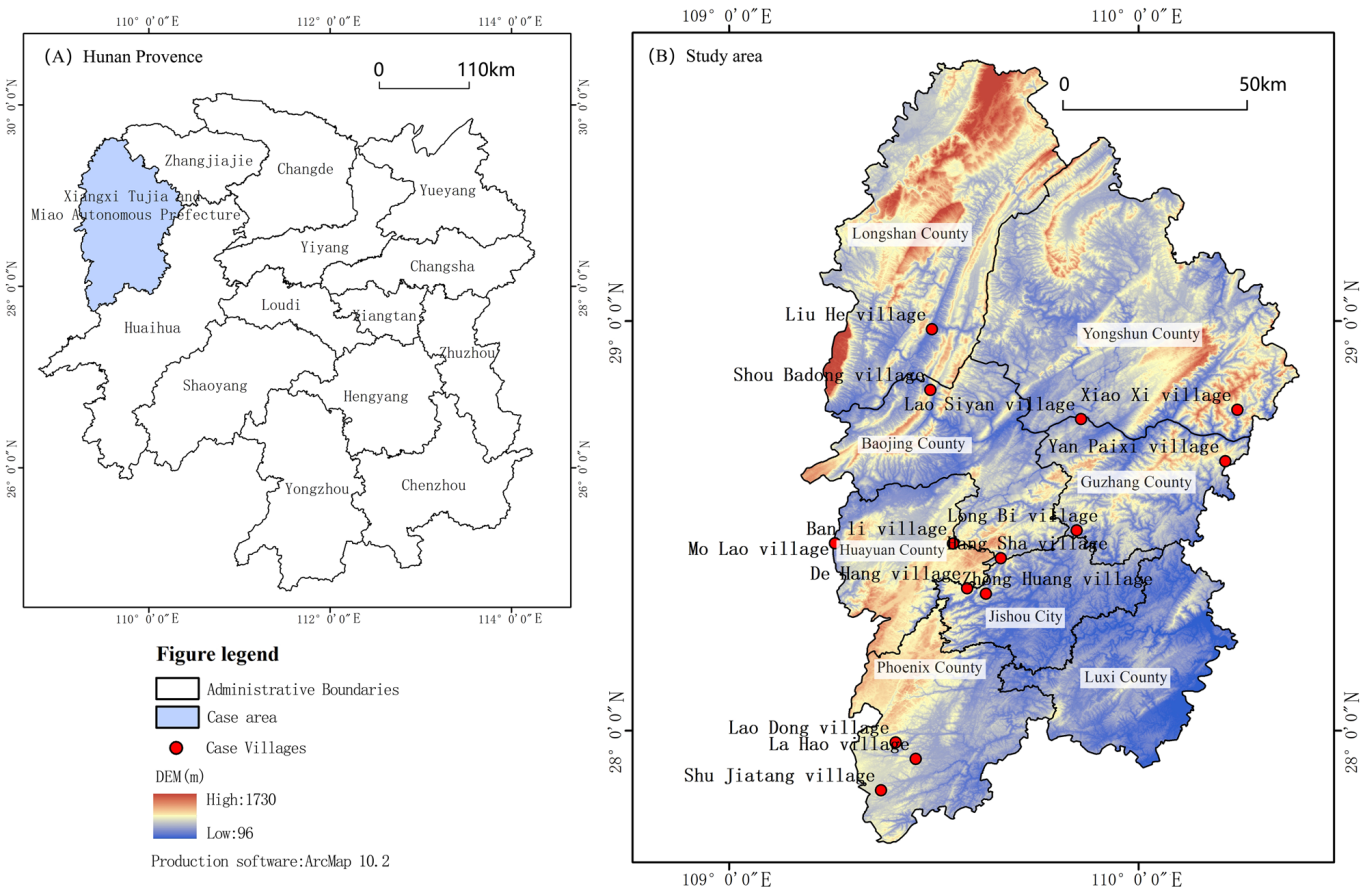
A map showing the study region (drawn by the authors using Arcgis10.2 software). (**a**) Location of the study area in Hunan Province; (**b**) The location of traditional villages in western Hunan. The source of DEM data of Hunan Province: http://www.gscloud.cn/sources/accessdata/305?pid=302; the source of spatial coordinates data of Chinese traditional villages: https://doi.org/10.3974/geodb.2018.04.06.V1.

Western Hunan, situated at a low latitude, experiences a humid subtropical monsoon climate. The traditional wooden structure of varying quality undergoes natural degradation or collapse due to severe weather conditions or age^45^, resulting in a ‘self-destructive’ landscape fragmentation. Concurrently, urban development brings about changes in lifestyle and values, making traditional buildings inadequate to meet the population’s demands for functionality and comfort. As a result, traditional architecture is gradually replaced by modern buildings, leading to a heterogeneous landscape in terms of style, height, structure, and other features. This shift triggers localized heterogeneous landscape fragmentation. A comprehensive assessment of the degree of fragmentation of different architectural landscape types will facilitate more effective management and restoration of local cultural heritage. To explore the fragmentation characteristics across different batches of architectural landscape types, this study selected 14 traditional Chinese villages in Western Hunan as case studies (Fig. 3). However, due to data acquisition challenges, three villages from the first batch—Shuang Feng, Lao Sicheng, and Jabala—were excluded.

### Data and data processing

Traditional village architecture can be analyzed in terms of architectural style, age, structure, quality, number of stories, roof style, and function, reflecting its purpose and characteristics. Following the index system for assessing fragmentation across various types of traditional village architectural landscapes (Table 2), and adhering to the principles of similarity, comprehensiveness, and data accessibility, this study employed architectural quality to gauge ‘self-damaging fragmentation,’ as well as the architectural style (overall style), architectural age (time), and the number of stories (height). In addition, the housing, urban, and rural construction departments of counties and districts with traditional villages are required to prepare and complete traditional village protection and development plans within one year of the announcement of the village's inclusion in the national protection list. For data consistency, this study utilized the Traditional Village Protection and Development Plan as the data source, which was prepared by the respective departments of counties and districts with traditional villages. These data were used to analyze the fragmentation characteristics of architectural landscape types in the first batches of traditional villages in Western Hunan following their inclusion in the national protection list.

Firstly, an analysis of landscape diversity was conducted, employing Eqs. (1), (2), and (3) to derive the relative evenness indices across the various dimensions of the architectural landscapes for these 14 villages; Eqs. (1), (4), and (5) were used to calculate the relative richness indices across different dimensions. Subsequently, landscape heterogeneity was examined, and the different dimensional heterogeneity indices of the architectural landscapes of these 14 villages were obtained using Eq. (6). Finally, drawing from the fragmentation evaluation criteria (Table 1), the degree of fragmentation of architectural landscape types in the first batch of villages was assessed with respect to both landscape diversity and heterogeneity.

## Results

### Uniformity characteristics of traditional village architectural landscapes

**Figure 4:**
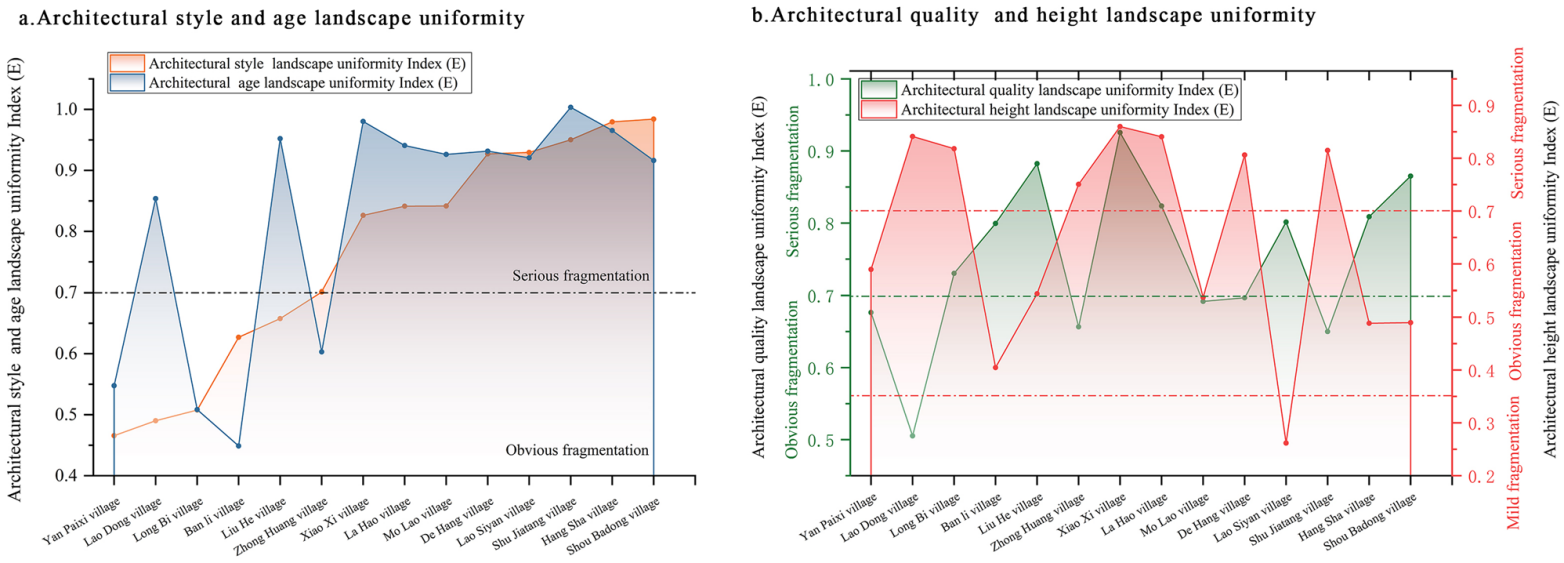
Relative uniformity index of architectural landscape in traditional villages in Xiangxi.

Regarding the uniformity of the landscape types, a higher relative uniformity index implies a greater inclination for an equivalent number of landscapes types. Based on the relative evenness index of the architectural landscape of the first batch of villages (Table 3, Fig. 4), the quantities of each type of landscape patch, including traditional architectural style, quality, and age, tended appeared nearly equal. The landscape complexity was high; signifying clear to severe fragmentation, with the architectural landscape’s height showing comparatively lower fragmentation. For the village of Yan Paixi, the architectural quality, style, age, and height were in the fragmentation stage, whereas those of La Hao and Xiao Xi were in severe fragmentation stages. Generally, villages exhibiting severe fragmentation had equivalent quantities for each type of landscape, whereas those with evident fragmentation had a certain primary landscape type and roughly the same number, albeit fewer, of the other landscape types.

**Table 3:** List of relative uniformity indices of architectural landscape in traditional villages.

Serial no.	Village name	Architectural style (E)	Architectural age (E)	Architectural quality (E)	Architectural floors (E)
1	Ban li village	0.6270	0.3989	0.7995	0.4047
2	De Hang village	0.9271	0.8816	0.6969	0.8061
3	Hang Sha village	0.9796	0.9154	0.8088	0.4885
4	La Hao village	0.8414	0.8908	0.8237	0.8405
5	Zhong Huang village	0.7012	0.5532	0.6566	0.7506
6	Lao Dong village	0.4902	0.8038	0.5053	0.8411
7	Lao Siyan village	0.9294	0.8707	0.8014	0.2619
8	Liu He village	0.6576	0.9021	0.8824	0.5443
9	Long Bi village	0.5077	0.4585	0.7302	0.8179
10	Yan Paixi village	0.4657	0.4977	0.6764	0.5896
11	Mo Lao village	0.8416	0.8762	0.6919	0.5375
12	Shou Badong village	0.9842	0.8662	0.8652	0.4898
13	Shu Jiatang village	0.9503	0.9535	0.6500	0.8148
14	Xiao Xi village	0.8263	0.9303	0.9256	0.8598

More specifically, (1) three categories of architectural quality landscapes exist: good, medium, and poor. Approximately 57.1% of the relative uniformity indices for traditional village architectural quality landscapes fell within the (0.7, 1] range indicating a relative similarity in the numbers of good, medium, and poor landscape types—a scenario representative of severe fragmentation. Approximately 42.9% were in the (0.35, 0.7] range, signifying that the numbers of good, medium, and poor landscape types were relatively balanced, correlating with evident fragmentation (Fig. 4b).

(2) Generally, there are four types of architectural landscapes: cultural preservation units, historic buildings, traditional buildings (including new style-coordinated buildings), and new style-uncoordinated buildings. The ratio of severe and evident architectural landscape fragmentation in the 14 villages resembled that for architectural quality landscape, demonstrating general consistency across the villages. For instance, the villages of Lao Dong and Yan Paixi exhibited evident fragmentation of the architectural landscape and landscape quality, whereas Lao Siyan, Shou Badong, Xiao Xi, La Hao, and Hang Sha showed severe fragmentation (Fig. 4a).

(3) Traditional villages feature four categories of architectural chronological landscapes: the Qing dynasty, the Republican period, the 1950s to the 1970s, the 1980s, and post-1980s. About 71.4% of the village building chronological landscapes were within the range (0.7, 1] in terms of the relative evenness index, indicating severe fragmentation and suggesting that at least three of the four chronologies had approximately the same number of buildings. Meanwhile, 28.6% were in the (0.35, 0.7] range with significant fragmentation, indicating that at least two of the four chronologies contained approximately the same number of buildings (Fig. 4a).

(4) There are three types of building height landscapes: one story, two stories, three stories, and more than three stories. The village of Lao Siyan was predominantly composed of one-story building landscapes, accounting for 93%, whereas two- and three-story buildings were less common, resulting in low fragmentation in the building height landscape. In the villages of Lao Dong, Xiao Xi, and another 50% of other villages, all three types of building heights represented a certain proportion of landscape complexity, with fragmentation being severe. In 42.9% of villages, such as Ban Li, there are primarily one- and two-story buildings and a few with over three stories, exhibiting evident fragmentation (Fig. 4b).

Figure 4 provides a visual representation of the relative uniformity index of the architectural landscape and diversity fragmentation characteristics of each traditional village in Western Hunan. Figure 4a shows the relative uniformity index of the architectural style and age. Figure 4b illustrates the relative uniformity index of the architectural quality and height.

### Richness characteristics of traditional village architectural landscapes

**Figure 5:**
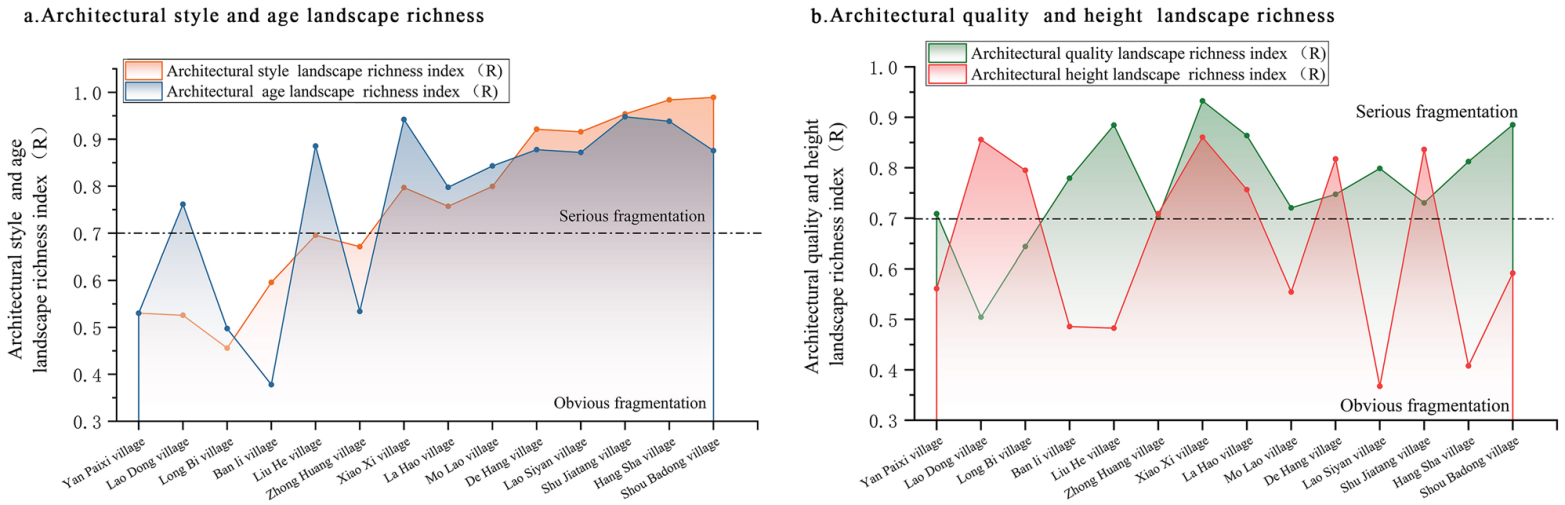
Relative richness index of architectural landscape in traditional villages in Xiangxi.

In terms of the richness of the landscape types, the greater the relative richness index, the richer the landscape type. According to the relative richness index of the architectural landscape of the studied villages (Table 4 and Fig. 5), there were three landscape types based on the quality and height of the traditional buildings and four based on the age and style of the buildings. The different dimensions of the architectural landscape types were relatively rich, landscape complexity was high, and most showed evident or severe fragmentation. The architectural quality, style, age, and height of Lao Hao, De Hang, Shu Jiatang, and Xiao Xi were severely fragmented. Generally, given that the landscape types are the same and the number of landscape types is equal, the richer the landscape type, the more severe the fragmentation.

**Table 4:** List of relative richness indices of architectural landscape in traditional villages.

Serial no.	Village name	Architectural style (R)	Architectural age (R)	Architectural quality (R)	Architectural floors (R)
1	Ban li village	0.5957	0.3781	0.7791	0.4858
2	De Hang village	0.9213	0.8778	0.7473	0.8174
3	Hang Sha village	0.9839	0.9382	0.8121	0.4078
4	La Hao village	0.7571	0.7976	0.8636	0.7565
5	Zhong Huang village	0.6711	0.5341	0.7015	0.7089
6	Lao Dong village	0.5258	0.7615	0.5044	0.8558
7	Lao Siyan village	0.9158	0.8719	0.7986	0.3675
8	Liu He village	0.6954	0.8854	0.8844	0.4827
9	Long Bi village	0.4558	0.4974	0.6438	0.7947
10	Yan Paixi village	0.5302	0.5303	0.7086	0.5611
11	Mo Lao village	0.7995	0.8431	0.7204	0.5544
12	Shou Badong village	0.9891	0.8757	0.8851	0.5917
13	Shu Jiatang village	0.9538	0.9478	0.7303	0.8361
14	Xiao Xi village	0.797	0.9421	0.9326	0.8604

More specifically, (1) there were three landscape types: good, medium, and poor architectural quality in all 14 traditional villages. Approximately 85.7% of these villages showed a relative richness index for architectural quality landscape in the (0.7, 1] range. The number of good, medium, and poor landscape types was relatively uniform, with rich landscape types and severe fragmentation. Villages with evident fragmentation accounted for 14.3% and were dominated by one type of architectural quality, while the others accounted for much less. For example, the villages of Lao Dong and Long Bi had 0.9% and 0.6% poor-quality buildings, respectively (Fig. 5b). (2) There were usually three or four types of architectural landscapes, of which 57.1% of the villages had a relative richness index in the (0.7, 1] range, showing severe fragmentation. In such villages, either the number of the three landscape types tends to be the same or the number of at least three of the four landscape types is relatively uniform. Approximately 42.8% of the villages were in the (0.35, 0.7] range, with evident fragmentation. This type of village was dominated by one type of architectural landscape, which usually accounted for over 75% (e.g., 75.2%, 80.2%, and 81.5% of the dominant landscape types observed in Long Bi, Lao Dong, and Yan Paixi, respectively) (Fig. 5a). (3) There are four types of landscapes in all villages in architectural chronology except Xiao Xi, which has three types. When the landscape types are consistent, the relative richness index is proportional to the relative evenness index. The higher the relative evenness index, the higher the relative richness index. Therefore, the degree of fragmentation in each village in the landscape richness index was consistent with that of the landscape evenness index (Fig. 5a). (4) In half of the villages, including Xiao Xi, Lao Dong, and Shu Jiatang, two or three building stories accounted for a higher proportion of landscape complexity, with severe fragmentation. Fifty percent of the villages, including Lao Siyan and Shou Badong, showed evident fragmentation, with buildings primarily comprising one or two stories. For example, no buildings had three or more stories in Lao Siyan or Shou Badong (Fig. 5b). Figure 5 depicts the relative richness index of the architectural landscape and the diversity fragmentation characteristics of each village in the traditional villages of Western Hunan. Figure 5a shows the relative richness index of architectural style and age, and Fig. 5b shows the relative richness index of architectural quality and height.

### Heterogeneous characteristics of the architectural landscape as a whole

**Figure 6:**
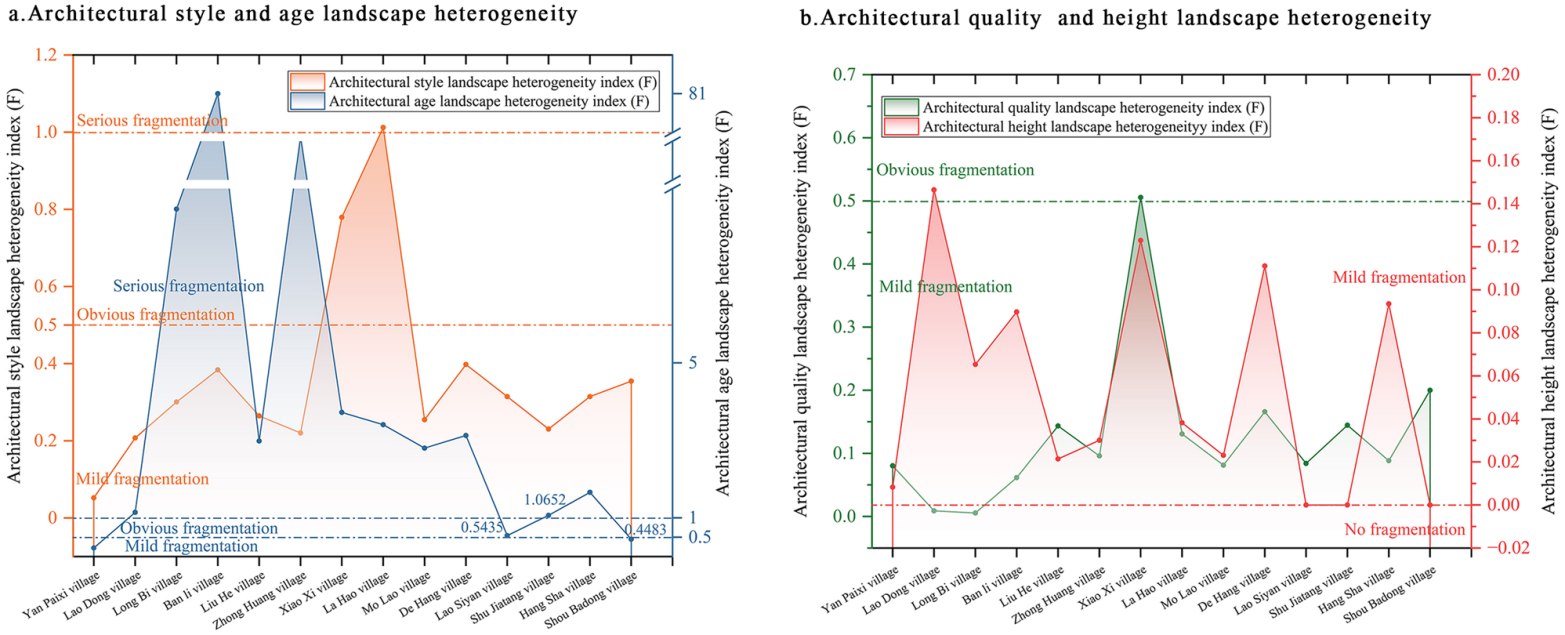
Architectural landscape heterogeneity index of traditional villages in Xiangxi.

Concerning the landscape heterogeneity index (Table 5, Fig. 6), the 14 traditional villages exhibited minimal fragmentation in relation to architectural quality, style, and height, with values of 92.9%, 85.7%, and 100%, respectively. This suggests that heterogeneous landscapes in these three dimensions have a negligible influence on traditional landscape fragmentation. Approximately 78.6% of the heterogeneity index of architectural age landscape in villages was in the (1, + ∞) range. In other words, the number of post-1950s buildings was more than double that of pre-1950s buildings. Thus, the heterogeneous landscape is much larger than the traditional landscape in the time dimension, leading to severe fragmentation. Despite the construction of many new buildings in traditional villages due to urbanization and rural tourism growth, these buildings harmonized with the traditional landscape and their building heights aligned with those of traditional buildings.

**Table 5:** List of architectural landscape heterogeneity indices of traditional villages.

Serial no.	Village name	Architectural style (F)	Architectural age (F)	Architectural quality (F)	Architectural floors (F)
1	Ban li village	0.3840	81.0000	0.0615	0.0897
2	De Hang village	0.3978	3.1270	0.1659	0.1111
3	Hang Sha village	0.3146	1.6591	0.0884	0.0935
4	La Hao village	1.0123	3.4054	0.1307	0.0382
5	Zhong Huang village	0.2205	15.1176	0.0960	0.0301
6	Lao Dong village	0.2074	1.1415	0.0089	0.1465
7	Lao Siyan village	0.3148	0.5435	0.0840	0.0000
8	Liu He village	0.2646	2.9833	0.1435	0.0214
9	Long Bi village	0.3007	8.9722	0.0056	0.0653
10	Yan Paixi village	0.0522	0.2222	0.0804	0.0083
11	Mo Lao village	0.2547	2.8000	0.0813	0.0231
12	Shou Badong village	0.3548	0.4483	0.2000	0.0000
13	Shu Jiatang village	0.2306	1.0652	0.1446	0.0000
14	Xiao Xi village	0.7792	3.7241	0.5055	0.1230

### Heterogeneous characteristics of architectural landscapes in different dimensions

Architectural landscape heterogeneity varies across different dimensions, with architectural age having more heterogeneous landscapes, architectural quality, style, and height have fewer.

Specifically (1) there was a ‘self-damaging’ fragmentation of architectural landscape quality, with 92.9% of villages in the (0, 0.5] range and 7.1% of villages in the (0.5, 1] range. In essence, most villages had fewer poor-quality buildings and few heterogeneous architectural landscapes due to natural damage or collapse. All villages displayed mild fragmentation, except for Xiao Xi, which showed evident fragmentation (Fig. 6b).

(2) As the most visually apparent dimension of ‘heterogeneous landscape fragmentation,’ architectural style exhibited three fragmentation states; however, the heterogeneity index for each village did not differ significantly in the mild fragmentation state. The architectural style heterogeneity index for 85.7% of the villages was in the (0, 0.5] range, and the number of modern buildings outnumbering traditional ones by more than double. Xiao Xi was fragmented, while La Hao showed severe architectural style fragmentation, with modern buildings is more than double that of traditional buildings (Fig. 6a).

(3) Building height is the vertical intuitive dimension of ‘heterogeneous landscape fragmentation,’ with fewer heterogeneous landscapes of three or more stories and no or light fragmentation. For instance, Lao Siyan, Shou Badong, and Shu Jiatang consisted of one- and two-story buildings with no heterogeneous landscape impact. The remaining villages displayed slight fragmentation, with a low heterogeneity index (Fig. 6b).

(4) The architectural age landscape represents ‘heterogeneous landscape fragmentation’ in the time dimension. The 14 traditional villages exhibited three stages: light, evident, and severe fragmentation. However, the heterogeneity index for each village varied significantly. Yan Paixi and Shou Badong were mildly fragmented with heterogeneity indices of 0.2222 and 0.4483, respectively, whereas Lao Siyan was fragmented with a heterogeneity index of 0.5435. Most villages exhibited a high heterogeneity index for architectural age and severe fragmentation, with the highest heterogeneity observed in Ban Li, reaching 81, followed by Zhong Huang at 15.1176 (Fig. 6a). Figure 6 illustrates the architectural landscape heterogeneity index of the traditional villages in western Hunan and the heterogeneous fragmentation characteristics of each village. Figure 6b shows the landscape heterogeneity index for architectural quality, and height and Fig. 6a shows the landscape heterogeneity index for architectural style and age.

## Discussion

Landscape heterogeneity and fragmentation are objective phenomena that are pervasive in cultural landscapes and represent contradictions that inevitably occur during rural modernization and development^46^. The degree of fragmentation of landscape types can reveal architectural landscape characteristics and the regional modernization process of traditional villages. Light fragmentation indicates the integrity and homogeneity of architectural landscape types and reflects a lower level of economic development. Conversely, high fragmentation reflects rapid economic development, leading to a significant deterioration in the integrity and homogeneity of architectural landscape types. Traditional village landscapes are not static but rather evolve over time through a process of ‘superimposition and accumulation’. These cultural landscapes ‘inherit’ their unique regional (historical, ethnic, etc.) traits while undergoing certain cultural ‘mutations’ to adapt to new environments. However, their overarching genetic traits remain consistent, with exceptions occurring only in rare instances of ‘mutations. This ensures the preservation of their regional individuality and ‘local’ cultural heritage^47^. Therefore, one should not blindly aim for a low fragmentation level when aiming to protect and develop traditional villages, as this could hinder sustainable development. From an architectural landscape perspective, the layout of new buildings should consider the need to minimize fragmentation of architectural style and building height landscapes to meet new demands. For those buildings contributing to heterogeneous landscape fragmentation, landscape components should be altered by repairing deteriorating buildings and modifying modern architectural styles to ensure the conservation and sustainable development of traditional culture.

This study adopts diversity and heterogeneity estimation methods from landscape ecology theory to quantify the fragmentation of traditional architectural landscape types. The results obtained are objective, scientifically sound, and adaptable. However, the validity of these measurements hinges on data reliability; hence, it is vital to scientifically discern each dimension of the architectural landscape type (three-level index) when quantifying the degree of architectural landscape fragmentation. Future studies should investigate the following aspects: (1) methods and systems to quantify architectural landscape fragmentation; (2) the evolution of architectural landscape fragmentation in traditional villages across different periods, to further understand the trend of architectural landscape fragmentation in different dimensions and thus support scientific governance and restoration; (3) the underlying causes and mechanisms of architectural landscape fragmentation in traditional villages; and (4) landscape fragmentation in general, which is the process by which landscapes transition from a homogeneous and cohesive state to a discontinuous and patchy mosaic. Studying the fragmentation of architectural landscape types can reveal heterogeneous landscape fragmentation, but an analysis of the spatial pattern fragmentation of architectural landscapes can expose varying spatial fragmentation patterns triggered by landscape diversity or heterogeneity. These distinct patterns signify an inclination towards village development to a certain extent. Therefore, it is crucial for future research to explore the spatial pattern fragmentation of architectural landscapes.

## Conclusions

1. Architectural landscape fragmentation, which involves the alteration and decay of architectural landscape types, combinations, and properties across space or time, falls into two categories: ‘self-damaging fragmentation’ and ‘heterogeneous fragmentation’. The fragmentation of architectural landscape types was assessed from two perspectives (landscape diversity and heterogeneity) and across seven dimensions (architectural style, building age, structure, quality, height, roof style, and function). The measure of landscape fragmentation from the diversity perspective indicates whether fragmentation exists in each dimension of the landscape type within traditional village architectural landscapes. Measures of landscape fragmentation based on the heterogeneity perspective indicate whether fragmentation occurs in each dimension of the landscape type due to heterogeneous landscapes in traditional village architecture.
2. From the viewpoint of landscape diversity, the first batch of villages showed a rich variety of landscape types across all architectural dimensions and high relative evenness indices, exhibiting either evident or severe fragmentation. However, there were fewer villages with the same architectural landscape undergoing the same stage of fragmentation across different dimensions, with the majority of villages experiencing the same stage of fragmentation across two or three dimensions. Given the unique nature of architectural landscapes, the fragmentation resulting from a variety of landscape types is not entirely negative. For example, a uniform building height does not necessarily lead to an appealing skyline, whereas an appropriate variety in building height can enhance the vertical visual experience. Another example can be seen in the varying periods of architectural landscapes in traditional villages, which signifies that villages are not static; rather, but have undergone growth and expansion.
3. From the perspective of landscape heterogeneity, 14 villages had low heterogeneity indices for architectural quality, height, and style. Even though there was a diverse range of architectural landscapes across the aforementioned three dimensions, the number of heterogeneous landscapes (poor architectural quality, buildings with three or more stories, and modern buildings) was low. Landscape fragmentation caused by heterogeneous landscapes was minimal, and 85.7% of the village architectural landscapes showed low fragmentation across all three dimensions. The heterogeneity index for architectural age landscapes was high, the number of heterogeneous landscapes (buildings from the 1950s or later) was high, the degree of landscape fragmentation due to heterogeneous landscapes in the time dimension was high, and buildings from the PRC era or earlier had severely deteriorated or collapsed, requiring urgent protection and renovation. Generally, landscape fragmentation does not contribute to the preservation and protection of traditional landscape, except for considerations related to building age.

## Data Availability

The datasets generated and/or analyzed in the current study can be made available from the corresponding author upon reasonable request.
